# Enterprise Adoption of Telehealth: An Academic Medical Center's Experience Utilizing the Telehealth Service Implementation Model

**DOI:** 10.1089/tmr.2021.0006

**Published:** 2021-06-03

**Authors:** Shawn Valenta, Jillian Harvey, Emily Sederstrom, Meghan Glanville, Tasia Walsh, Dee Ford

**Affiliations:** ^1^ Wellpath, Nashville, Tennessee, USA.; ^2^ Department of Healthcare Leadership & Management and Medical University of South Carolina, Charleston, South Carolina, USA.; ^3^ Clinical Strategy and Integration, Oklahoma University Health, Oklahoma City, Oklahoma, USA.; ^4^ Center for Telehealth, Medical University of South Carolina, Charleston, South Carolina, USA.; ^5^ Department of Pulmonary and Critical Care Medicine, Medical University of South Carolina, Charleston, South Carolina, USA.

**Keywords:** telehealth, implementation, service development, operations

## Abstract

There are numerous challenges to developing and sustaining successful telehealth services and a paucity of guiding frameworks to inform telehealth strategy, design, and ongoing operations. The framework Telehealth Service Implementation Model (TSIM)™ was developed to provide a guiding telehealth framework that enables grassroots innovations and accounts for the many factors and domains necessary for successful telehealth service development, implementation, and sustainment. TSIM includes six phases: (1) Pipeline, (2) Strategy, (3) Development, (4) Implementation, (5) Operations, and (6) Continuous Quality Improvement. TSIM provides common terminology for improved team coordination, checkpoints, and milestones to facilitate scaling telehealth services, and a process to get stalled services back on track. TSIM provides an invaluable framework to assist organizations in developing a strategic vision for telehealth services, designing telehealth services enabled for success, and monitoring for high quality and high reliability.

## Introduction

In addition to being associated with significant patient and provider satisfaction,^1,2^ telehealth can provide highly successful clinical and educational services at a distance. Telehealth has shown to be instrumental in maintaining access to care during the COVID-19 social distancing restrictions.^3^

Yet substantial barriers to implementation persist. Telehealth fundamentally challenges the traditional roles, processes, and norms of regionalized health care delivery, and this disruptive quality is only one of the barriers to the development and sustainability of services.^4,5^ Other barriers include technical, financial, regulatory, and administrative burdens. In addition, lack of adequate planning before implementation is a commonly reported issue.^6–8^ Without improving implementation processes, the growth in demand may reinforce a cycle of developing unstainable telehealth services.

### Intervention setting and history

**FIG. 1.**
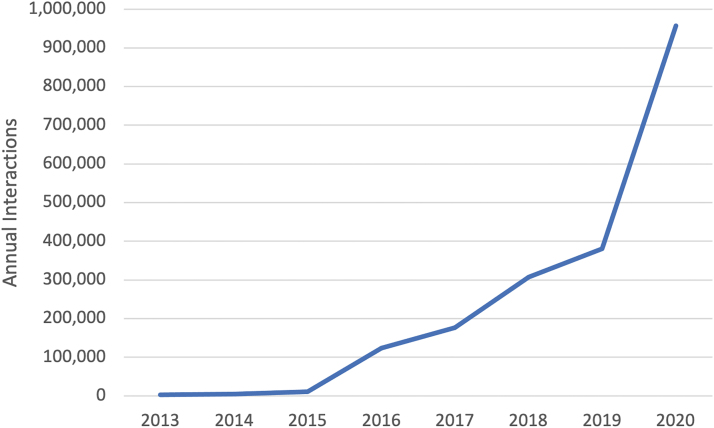
Annual telehealth interactions 2013–2020.

The Medical University of South Carolina (MUSC) is a federally recognized Telehealth Center of Excellence with extensive breadth and depth in telehealth program development, implementation, and evaluation. The MUSC Center for Telehealth (Center) was established in 2013 and built upon a legacy dating back to 2005 of providing telehealth services for maternal fetal medicine, telestroke, and critical care. MUSC presently offers 80 unique telehealth services at >300 clinical sites across 46 SC counties. Annual telehealth interactions grew from 3,091 in 2013 to nearly 1 million within 7 years (Fig. 1) and 78% of MUSC's services are to fully or partially medically underserved SC counties.

MUSC's information solutions department implemented an institution-wide information technology (IT) service management initiative based on the Information Technology Infrastructure Library (ITIL^®^) framework.^9^ This initiative was aimed at improving the overall delivery of IT services. ITIL provides best-practice guidance for the development, implementation, and ongoing support of IT services, and it has been adopted globally to support IT services across numerous industries. Through its observations of ITIL's impact on IT management, the Center saw the benefits of using a guiding framework, standardized processes, and a shared nomenclature for delivering services. It leveraged its vast experience in developing telehealth services to create a telehealth implementation model that established a common nomenclature, identified standardized processes, and provided a framework to understand the Center's strengths, weaknesses, and gaps to developing and managing their telehealth services.

A core goal of developing Telehealth Service Implementation Model (TSIM™) was to think beyond the dogma that telehealth should “replicate care over distance,” and instead, the Center sought to innovate telehealth to improve the efficiency and/or effectiveness of care.^10,11^ To initiate the creation of a telehealth framework, the Center administrator hosted a full-day retreat to discuss key concepts and processes that would be required for a telehealth framework. This included looking at gaps in existing frameworks and understanding what mission critical areas needed to be included in a telehealth framework. Finally, informed by these results, the Center administrator created the initial structure for our telehealth-specific framework, which has continued to evolve since 2017.

Although MUSC's framework was created out of a necessity to accelerate enterprise-wide adoption of telehealth, its guiding structure can be applied to the development of any telehealth service. We discuss the phases of TSIM, lessons learned through real-world vignettes, and describe how a guiding framework could have mitigated our challenges.

## TSIM Overview

**FIG. 2.**
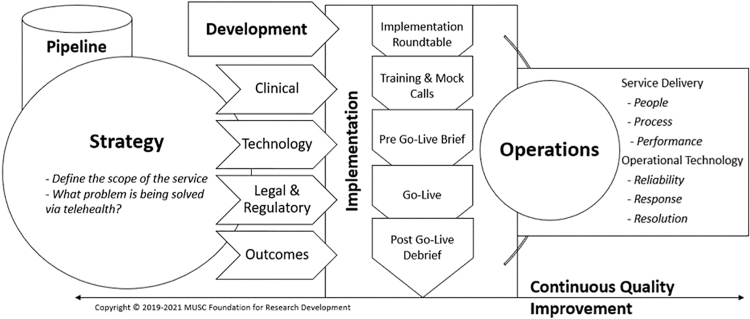
TSIM phases and related domains. TSIM, Telehealth Service Implementation Model.

The TSIM framework provides common terminology and creates standardized processes to address telehealth-specific issues. TSIM includes six phases: (1) Pipeline, (2) Strategy, (3) Development, (4) Implementation, (5) Operations, and (6) Continuous Quality Improvement (CQI). Each phase has domains with prespecified goals and benchmarks that must be achieved before a service advances to the next phase. Figure 2 provides a breakdown of key domains within each TSIM phase. Ultimately, TSIM enables proactive recognition of program strengths, weaknesses, and gaps in service development/management. TSIM also inspired a reorganization of Center personnel, and key roles now support the major TSIM phases. Before TSIM, telehealth services were designed vertically, often with a single person responsible for all activities associated with the telehealth service (training, scheduling, etc.). We now utilize a horizontal organizational structure and cross-train personnel within their TSIM phases.

## Pipeline

Innovation often comes from grassroots efforts. Thus, it is important to provide a forum for new telehealth service requests. The TSIM Pipeline organizes and manages the flow of service development requests. This has enabled ongoing innovation while simultaneously mitigating the risk of siloed programs untethered from organizational telehealth infrastructure. Pipeline also reduces duplication of efforts and prevents resource allocation to one-off projects, which are not aligned with organizational priorities.

Stakeholders can submit an electronic intake form through e-mail, which is ultimately reviewed by the Center for Telehealth Leadership. A telehealth coordinator is assigned to the request and collects additional documentation related to the purpose and support for the service. Next, a leadership team, including the Executive Medical Director and Administrator, explores the high-level description of the program and assesses the effort and resources necessary to implement the service, the estimated impact, and overall mission alignment. This analysis informs one of three status decisions: (1) No-Go; (2) On-Hold; or (3) Move to Strategy. If a service is determined a “No-Go,” the rationale is documented and shared with the requesting stakeholder. Documentation of “No-Go” decisions are essential to prevent repeated reworking, as telehealth service ideas periodically resurface. Ideas and pet projects that implement technology just for the sake of being novel are deemed “No-Go.” A service that has clear purpose is aligned with the organizational mission, has strong stakeholder support, and an appropriate technology generally moves to the Strategy phase.

### Avoiding duplicate services: Pipeline phase

Before the implementation of TSIM, nephrology and family medicine stakeholders-initiated work to develop an app to identify patients at-risk for chronic kidney disease and connect the patients with providers. The telehealth coordinator spent numerous hours meeting to vet ideas with stakeholders and developing a scope of work. Ultimately, it was revealed that similar technology was already being used and the service was duplicative. The processes conducted in the Pipeline phase are designed to catch such issues before investing resources.

## Strategy

Strategy is the starting point of a telehealth service development. The accompanying activities establish the scope, intended deliverables, and organizational means to provide the service. Strategy frames the design and approach to ensure strategic alignment and can account for many of the complex factors that may impede the success of the telehealth service (e.g., change management, economic feasibility, and legislative policies). The telehealth coordinator works collaboratively with the provider champion and administrative personnel to develop the business plan for the service, including an assessment of the key stakeholders, pro forma including expected patient volume, relative value units, and information on reimbursement mechanism(s). In addition, program success metrics are established to focus on utilization, quality, patient and provider experience, technical reliability, and cost-effectiveness. Three key benchmarks must be achieved for a service to be prioritized for subsequent development. First, the telehealth service must have a clearly defined goal and measures to characterize success. The foundational question is “what problem is being solved via telehealth?” Second, an indisputable link must be made between the technology and how it will enhance the efficiency and/or effectiveness of patient care. Finally, a standardized telehealth scoring tool is used to place a priority score on the service, and an assessment is made on the impact of the service on five stakeholder groups: (1) patients, (2) referring providers, (3) consulting providers, (4) payers, and (5) the health system. If a service is determined to have an overall negative impact on any of these key stakeholder groups, our experience has shown that the service will not be able to sustain at scale and a resolution must be identified. Before advancing the service to Development, the coordinator circulates the completed intake form internally within the Center to apprise the broader team of the new service.

### Understanding stakeholder demand: strategy phase

The Telehealth Specialty Service was designed to provide access to an array of subspecialists in rural primary care offices. Access to specialty care was identified as a major need in rural areas and substantial resources were invested to develop >20 telehealth specialty services, but in the first 5 years of operations, nutrition and psychiatry represented >84% of the 1923 telehealth consultations. The lack of demand for the other service options created challenges to provider proficiency and long-term sustainability. The service strategy was redesigned through TSIM to focus exclusively on outpatient nutrition and psychiatry. Since the strategy redesign, the overall service utilization has increased by 92%. A key lesson learned in this situation is the clear difference between “demand” and “need.” Although data may indicate the need for particular telehealth services, unless all stakeholders agree on the demand, it will never reach meaningful utilization.

## Development

**FIG. 3.**
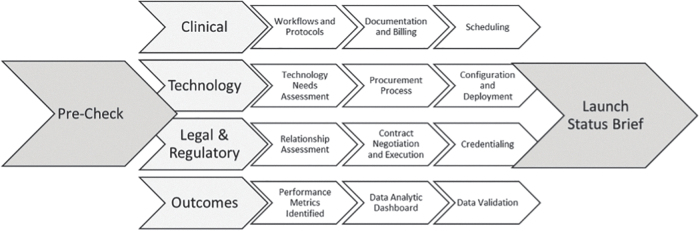
Detailed process map for TSIM Development Phase. © 2019–2021 MUSC Foundation for Research Development, All Rights Reserved. MUSC, Medical University of South Carolina.

Strategy is put into action during the Development phase. A detailed process map of Development is provided in Figure 3. A site readiness assessment is conducted to engage the site coordinator and evaluate the technical requirements. Development is organized by clinical, technology, legal and regulatory, and outcomes categories and is typically the most time-consuming TSIM phase. After a site assessment, the operational steps to develop the clinical pathway include a service precheck, workflow development, technical system(s) development, and scheduling protocols. Service prechecks include assessing the need for technical changes and electronic health record (EHR) modifications both of which can add substantial time to the development process. Technical system development includes establishing workflows for patient scheduling, registration, documentation, and billing. This step may include system upgrades and changes for scheduling, EHR documentation, user rights, and billing compliance. Workflow development includes both clinical and operational workflows. An important clinical assessment is to evaluate the service's impact on staffing and resources. After the site readiness assessment and concurrent with the clinical pathway development, tasks are completed for functions that support the clinical service, including (1) legal and regulatory, (2) procurement and asset tracking of technology, and (3) completion of an outcomes and reporting plan. All of these tasks inform the final step in Development, the launch status brief. During the launch status brief, internal stakeholders vet the near-final service design and confirm a tentative “go-live.”

A risk of the Development phase is that key stakeholders will be either overburdened or conversely excluded from key decisions. Therefore, quality assurance checks are held to ensure that all stakeholders are comfortable with the new service and workflows by being consulted and/or informed on key steps. A timeline for training and service go live are developed. In our experience, the most common bottlenecks are contracting with external sites, provider credentialing, and technology implementation—especially if modifications to an EHR are necessary.

### Removing bottlenecks: Development phase

Common telehealth implementation bottlenecks include the contractual process with new sites and modifications to technical systems such as the EHR. Often, the groups involved in these steps are outside the purview of our Center. Initially, our EHR did not have a standard pathway by which to designate and/or recognize a telehealth patient encounter. Thus, telehealth patients were attributed to various ambulatory sites resulting in numerous issues both internally and for partner sites. In our experience, it is imperative to have a methodical approach for submitting new telehealth sites, services, and providers to ensure EHR system changes are completed in a standardized, compliant, and timely manner. Any delays for new services are problematic as lost time can result in reduced stakeholder engagement and the need to repeat or update prior work.

## Implementation

Implementation converts the program's Strategy and Development into a functional service and includes an implementation roundtable, training and mock calls, a pre-go-live brief, go-live, and a post-go-live debrief. During the implementation roundtable, providers and other key clinical personnel are educated on any necessary technical, workflow, and regulatory issues. This might include training in the roles and limitations of telepresenters, technical walkthroughs, and training among external staff at referring sites. Mock calls are conducted to test the internal and external workflows. A pre-go-live brief is the final check to ensure all stakeholders understand their roles and responsibilities before go-live. During go-live, the service is operational and is followed prospectively to monitor for any incidents that may impact quality and effectiveness. Debriefs are utilized to review any opportunities for improvement. Telehealth services remain in Implementation for variable time periods to ensure that enhanced support for technology and operational needs are available during the early stages of telehealth service delivery. This enhanced support is imperative to maintain relationships and engagement with provider champions and remote sites. Mistakes that are not quickly resolved during Implementation can have lasting adverse impact on the reputation of the service and ultimately on the sustainability of the program. After meeting a predetermined measure of success (e.g., patient volume) the service moves into the Operations phase. Services in the Implementation phase that are not meeting goals are continually revisited and root cause analysis through CQI are conducted.^12^

### Getting it right: Implementation phase

After going live with a new infectious disease service, the initial feedback from the consulting providers was not favorable. The post-go-live debrief uncovered a collection of contributing factors that were not within the original scope of the service but deemed necessary after experience with the initial acute care consults. Since these elements were not built within Development, a leadership task force was quickly assembled and the lead service coordinator was assigned to work daily with the clinical champion on a task list that included workflow, EHR, and technical adjustments. Without having a systematic way to quickly review and address post-go-live issues, telehealth services risk provider disengagement, and subsequent unsustainability.

## Operations

During the Operations phase, the service is in a live environment, patient encounters occur, and efforts are focused on service delivery and operational technology management. Service delivery includes the management of the day-to-day operations of the delivery of high-quality telehealth services that continue to improve quality care and the patient and provider experience. Key processes include customer engagement, service proficiency, service quality, provider performance, and business sustainability. Operational technology management handles the day-to-day operations of the technical infrastructure, systems, and devices to ensure services maintain high reliability. Key processes of operational technology management include infrastructure reliability, incident response, and technical resolution management. Services in Operations must have a process to identify and address “incidents,” defined as any unplanned event that could negatively impact normal operations. Revisiting mock calls and trainings is necessary to address deviation and/or modifications of workflows or as refreshers for low volume services or providers. During Operations, continuous assessment of service goals, monthly performance indicators, quarterly optimization, and annual reporting are used to identify services that should be archived or optimized through the CQI phase.

### Technical resolution support: Operations phase

A chronic disease management program uses the combination of nurse case management and remote patient monitoring to facilitate improvements in glycemic and blood pressure control in rural and vulnerable populations. The original version of the program was slow to be adopted because of technology challenges that resulted in an average data transmission success rate of <50%. The effort in managing the technological incidents limited the program's monitoring capabilities to no more than 100 patients at a time. The technology problem led to exploring new devices and implementing a new cellular solution. Since the change, data transmission success rates are now averaging 80–90%, and the program is monitoring >600 patients at any one time.

## Continuous Quality Improvement

Plans for service evaluation are created during Development to ensure data can be reported in a format that facilitates quality improvement and data-driven decision making. Key performance indicators focus on utilization, quality of care delivery, patient and provider experience, technical reliability, and business sustainability. CQI responsibilities are fused into day-to-day management and data are utilized to inform improvements to the service delivery and program outcomes. Within CQI, traditional quality improvement activities take place to improve processes and outcomes. In some cases, it may be determined that a service needs to move back to a prior TSIM phase and work through certain activities again.

The CQI team monitors four cross-cutting domains for all services. First, the technical reliability of the service is continuously monitored to ensure the service technology is sound, and interactions are not dropped. Second, quality metrics are captured to assess the efficiency of care delivery. The final two CQI domains are patient and provider experience with the telehealth service. In addition to the four TSIM CQI measures, each service has individualized guideline-based clinical quality metrics that are equivalent to in-person care. It is also recommended that programs work toward inclusion of all the NQF Measurement Framework Domains (Access to Care, Financial Impact/Cost, Experience, and Effectiveness).^13^

### Enhancing the effectiveness and efficiency: CQI Phase

The Continuous Virtual Monitoring program enables “telesitters” to provide 24/7 supervision of patients who are at increased risk of falls or injury. Through the CQI process, it was identified that communication of safety concerns and the level of urgency were not always clear between telesitters and nursing staff. Therefore, a consistent nomenclature was developed to communicate dangerous patient behaviors. In addition, a decision tree for notification was implemented in which certain safety concerns are sent directly to the unit charge nurse. Specifically, the telesitter will immediately escalate to the charge nurse self-harm, violent behavior, or sexual behavior.

Systematic barriers to telehealth success are well described, including technology, change management, organizational structures, economic feasibility, user-friendliness, evaluation, evidence, legislation, policies, and governance.^5,14,15^ Although there are existing telehealth implementation models available, these are often theoretical and lack strategic and operational guidance. Before the development of TSIM, we experienced common telehealth pitfalls, including investing in telehealth technology that is never realized into a viable program, establishing telehealth programs without necessary provider engagement, setting up programs without a clear pathway from the technology to the patient, and establishing programs untethered from larger organizational strategies.^15^ Owing to the breadth and depth of MUSC's telehealth services and rapid program expansion, we encountered the need for an overarching framework that could be applied to any clinical service and any telehealth modality/technology. TSIM was developed to provide a guiding telehealth framework that enables grassroots innovations and accounts for the many factors and domains necessary for successful telehealth service development, implementation, and sustainment. Our Center's early years were characterized by an *ad hoc* approach, which was not a sustainable method. For example, after TSIM implementation, all telehealth services were reviewed and ∼20% were archived due to inadequate progress. Other services were consolidated within larger programs to avoid duplication of efforts. In review of failed services, poor strategic planning, inadequate financial considerations, and rushing key steps were especially notable failure points that TSIM addresses in Strategy and Development.

The utilization of telehealth within the United States and other countries continues to grow. However, barriers to implementation are common. We found TSIM helps address three of the most common barriers, including clinical appropriateness of the service, physician buy-in, and leadership support.^16^ Although TSIM has mechanisms to understand the program costs and reimbursement criteria during the strategy phase, more work is needed to fully resolve the biggest barrier to telehealth adoption, lack of reimbursement. Recently, COVID-19 led to the rapid implementation of telehealth services in many health systems.^4^ We believe a structured framework to developing and implementing telehealth is essential during times of rapid change and longer-term transformational efforts, as a way to ensure sound processes are put in place and in monitoring service quality.

### Future of TSIM

Through strategic partnerships, the Center is collaborating with external entities with global experience in software development and curriculum creation to help scale TSIM and the lessons learned at the Center to a broader audience. Health care systems outside of the Center are already beginning to apply the TSIM framework within their institutions, and their adoption of TSIM will continue to fuel its maturation. The overall goal of these new partnerships is to catalyze the adoption of telehealth and drive the efficient and effective development, implementation and long-term sustainability of high-quality telehealth services. Future research should examine the utilization of TSIM across different settings and under different contexts. In addition, next steps include assessment of program metrics to better understand the relationship between TSIM and telehealth service efficiency and sustainability.

## Conclusion

TSIM provides checkpoints and milestones to facilitate provider engagement, user-friendliness, pathways to scale telehealth services, and optimize administrative and technical support. We provide this overview of TSIM in hopes that other health care leaders can leverage the experience of the Center and utilize a guiding framework, such as TSIM, to accelerate the adoption of telehealth services at their organization.

## Abbreviations Used

CQI: Continuous Quality Improvement
EHR: electronic health record
IT: information technology
ITIL: Information Technology Infrastructure Library
MUSC: Medical University of South Carolina
TSIM: Telehealth Service Implementation Model
